# Case of Excision of the Scapula

**Published:** 1853-05

**Authors:** S. D. Gross

**Affiliations:** Professor of Surgery in the Medical Department of the University of Louisville


### SELECTIONS

FROM

### FOREIGN AND HOME MEDICAL JOURNALS.

---

*Case of Excision of the Scapula.* By S. D. Gross, M. D., Professor of Surgery in the Medical Department of the University of Louisville.—Mathew Gracey, aged forty, a gentleman of small stature and delicate frame, of a nervous temperament, and a merchant by occupation, of Eddyville, Caldwell County, Kentucky, applied to me in September, 1850, on account of an enormous tumour of the right shoulder, which he had first perceived about nine years before. His general health had always been good, and was so at the time of his visit. He was not conscious that his shoulder had ever received any injury, and no disease of a similar kind ever existed in any member of his family.

The tumor was fifteen inches in its vertical diameter, and fifteen inches and a half in the transverse. Its surface was perfectly smooth, except at one point, a little above its centre, where it was slightly tuberculated. It was hard and incompressible in its entire extent; the skin was perfectly sound, and there was no enlargement of the subcutaneous veins. By taking hold of it, it could be moved about from side to side, and lifted somewhat from the subjacent parts. Its superior limit corresponded with the shoulder-joint, anteriorly it projected into the axilla, and below it reached as far down as the ninth rib. Pressure upon the tumor produced no pain or uneasiness, except at its upper extremity, near the edge of the trapezius, where it was, and had been, quite tender for some time. During the last three months, the swelling has been the seat of a dull, heavy, aching pain, extending up the neck, and down the right arm as low as the elbow, most distressing at night, and always aggravated by the recumbent posture, probably in consequence of the pressure of the bed. The patient was unable to raise the corresponding limb without the aid of the sound one, and the shoulder was sensibly dragged forward towards the axilla by the weight of the tumor. The outline of the scapula could be distinctly traced only behind and above.

The tumor had increased rapidly within the last twelve months, and had, in great measure, disqualified Mr. Gracey for the active duties of his vocation. Four years ago, when he first consulted me respecting it, and when I strongly advised an operation for its removal, it was scarcely the size of a large fist, and entirely free from pain and inconvenience.

Having prepared my patient's system by purgatives, rest, and a properly regulated diet, I performed the operation of excision, on the 26th of September, in the presence of my friend Dr. James Johnston, Professors Miller and Rogers, and Drs. Colescott, Raphael, Thomson, Trabue, Clark, Washington, and Murray. A full dose of chloroform having been administered, an incision sixteen inches in length, was made from the superior angle of the scapula to the inferior extremity of the tumor, its direction being obliquely downwards and inwards. Another, beginning about five inches below the upper end of the first, and terminating about the same distance from its lower end, was then carried, in a curvilinear direction, so as to include the small oval flap of skin with the tubercle, previously alluded to, in its centre. The integuments, which were exceedingly dense and thick, especially at the superior part of the tumor, were then dissected off from the surface of the morbid growth, first towards the spine, and then towards the axilla. Having detached the elevator and trapezius muscles, I sawed through the acromion process of the scapula just behind the clavicle, and then divided the broad dorsal and anterior serrated muscles. Carrying my fingers next underneath the tumor, and raising it up, I severed its connections with the ribs, cut the deltoid and other muscles of the arm, sawed the neck of the scapula, and thus removed the entire mass with comparatively little difficulty.

Several vessels were divided in the early stage of the operation, at the posterior and middle part of the tumor; but these were easily controlled by the fingers of my assistants. Several arteries near the neck of the bone bled so freely as to demand the ligature after the removal of the morbid growth. About twenty-four ounces of blood were lost. The patient became very faint towards the close of the operation, and cordials were necessary to revive him.

The immense wound thus produced was dressed with three interrupted sutures and adhesive strips, and supported by a compress and a broad body bandage. The patient was placed in bed, and immediately took one grain of morphia.

At 4 o'clock in the afternoon there was a slight oozing of blood from the wound, and the patient complained of the tightness of the dressings, which, however, were found to be sufficiently loose. He had taken half a grain more of morphia, had slept some, and was free from pain: the pulse was 76, and of good volume; and there was no nausea, urgent thirst, or restlessness. On the following evening, September 27, the patient having slight traumatic fever, was ordered ten grains of calomel with one of opium and one of ipecacuanha, to be followed in the morning by castor-oil.

No untoward symptoms of any kind occured after the operation; nearly the whole wound healed by the first intention; and. at the end of three weeks, my patient went home with every prospect of a long and prosperous life. In descending the Ohio River, however, which was at that time exceedingly low, and which caused his detention upon the way for nearly a fortnight, he took a severe cold, from the effects of which he never completely recovered. A harassing cough set in accompanied by all the symptoms of pleuro-pneumonia, which were followed, about the middle of December, by those of hectic fever, under which he gradually sank, three months after the operation.

Soon after Mr. Gracey reached home, a small fungus was no iced in the course of the lower angle of the wound, which gradually increased in size, was very red and painful, occasionally bled a little, and obstinately resisted every effort that was made to heal it by his physicians, Drs. Carson and Champion. The latter of these gentlemen writes thus in relation to my patient's general illness:—

"When Mr. Gracey got home, he had a severe cough, which he thought depended upon cold he had caught in descending the Ohio River. The cough continued to increase, becoming more and more annoying, and was soon followed by severe pains, of a pleuritic character, in the chest. These pains frequently lasted for hours at a time, and generally required morphia for their relief; in the intervals, the lungs were always much embarrassed, the respiration being quick and hurried. His suffering, in fact, was constant; he had no appetite, an could not sleep, except when under the influence of anodynes. He became excessively emaciated, and a few days before he expired his reason gave way."

It is to be regretted that no *post-mortem* examination was made, as this would, at once, have revealed the true state of the thoracic viscera, and shown whether there was any cancerous disease at the side of the fungus, or elsewhere. If Mr. Gracey really had pleuro-pneumonia, as was supposed by his attendants, and if this disease, contracted while he was detained on board a steamboat, was neglected, it is not improbable that the fungous growth was not of a specific character, but the effect merely of ordinary unhealthy action.

The neck and glenoid cavity of the scapula were perfectly sound, as were also the various muscles connected with the tumor, the posterior surface of which was covered by the spinate muscles, in a state of great expansion and attenuation. The morbid mass weighed seven pounds and two ounces immediately after its removal, and belongs to the kind of structure, usually, though vaguely, denominated osteo-sarcomatous.

